# Effectiveness of the holistic primary school-based intervention MindMatters: study protocol for a cluster-randomised controlled trial

**DOI:** 10.1186/s13063-023-07731-0

**Published:** 2023-11-08

**Authors:** Lisa Fischer, Katharina Liegmann, Matthis Morgenstern, Kevin Dadaczynski

**Affiliations:** 1 Department of Health Sciences, Fulda University of Applied Sciences, Fulda, Germany; 2 Institute for Therapy and Health Research, Kiel, Germany; 3 Center for Applied Health Sciences, Leuphana University Lueneburg, Lueneburg, Germany

**Keywords:** Mental health, Whole-school interventions, Behavioural problems, Social-emotional skills, Academic performance, CRCT

## Abstract

**Background:**

The prevalence of mental health problems in childhood and adolescence has increased significantly, not least due to the COVID-19 pandemic in Germany and other countries worldwide. Although holistic school interventions to promote mental health and prevent mental health problems are considered promising, there is currently uncertainty about their effectiveness due to evaluation studies with heterogeneous methodological quality. This paper presents the study protocol for the evaluation of the primary school module of MindMatters.

**Methods:**

As part of a universal mental health intervention, the MindMatters primary school module ‘Learning Together with Emotions’ aims to promote social-emotional learning (SEL) in the classroom across five skill areas. In addition to classroom activities, the intervention includes a school development module to help primary schools create structures and processes to maintain and promote mental health. To evaluate the effectiveness of the intervention, a two-arm cluster-randomised controlled trial will be conducted, including schools implementing MindMatters over a 12-month period and a control group with no access to the intervention. Data will be collected before and 18 months after initiation of the intervention. Controlled for baseline conditions, multilevel regression analysis will be used to examine primary intervention outcomes at the pupil level (i.e. reductions in mental and behavioural problems). Further mediation and moderation analyses will examine whether proximal outcomes predict changes in mental health outcomes and whether school-level factors influence the effectiveness of the intervention.

**Discussion:**

This study will contribute to strengthen the evidence base for holistic school (mental) health promotion interventions using a study design with high internal validity. Based on an intervention model, the results will not only provide insights into the relationship between proximal and distal outcomes, but will also allow conclusions to be drawn about how the implementation of the intervention affects its effectiveness. Finally, the findings also address the question of whether improved mental health has a positive effect on primary school pupils’ academic performance.

**Trial registration:**

German Clinical Trials Register DRKS00023762. Registered on 5 January 2021.

**Supplementary Information:**

The online version contains supplementary material available at 10.1186/s13063-023-07731-0.

## Introduction

Although childhood and adolescence are considered comparatively healthy periods of life, especially in industrialised countries, researchers point to an epidemiological shift that places greater emphasis on non-communicable diseases (NCDs) such as mental health problems [1]. Indeed, an analysis of the Global Burden of Disease (GBD) 2015 study revealed that mental disorders were the second leading cause of disability-adjusted life years (DALY) among children aged 5–14 years in America and Europe [2]. A meta-analysis of 41 studies from 27 countries estimated a global pooled prevalence of mental disorders in children and adolescents of 13.4%, with the highest prevalence rates found for anxiety and disruptive disorders [3]. Summarising studies using the General Health Questionnaire (GHQ-12), a recent review found a global prevalence of common mental disorders of 25 to 31% depending on the cut-off value used [4]. However, the mental health of young people has deteriorated since the COVID-19 pandemic. In their recent rapid review, Schlack et al. [5] found evidence of significant increases in the proportion of children and adolescents with lower quality of life, higher levels of stress or more symptoms of specific mental disorders, particularly in the early phase of the pandemic. Results from the German longitudinal COPSY study indicate an increase in overall mental health problems from 18% (pre-pandemic) to 29% (mid-2020) to 31% (early 2021), which remained at a high level in the last wave (end 2021, [6]).

Based on these epidemiological findings, there is a strong need for interventions beginning in early childhood before the onset of mental health problems. Schools have long been identified as a key setting for (mental) health promotion and prevention for several reasons. First, it is argued that because of compulsory schooling, a large proportion of children and adolescents, regardless of their social, economic and cultural backgrounds, can be easily reached by school-based interventions. Second, schools are not only places that provide access to young people; they can also influence pupils’ mental health through environmental factors. Although the scientific evidence is heterogeneous, studies suggest that negative classroom learning environments (e.g. classrooms with fewer material resources and whose teachers receive less respect from colleagues) and low school climate (teacher ratings) are associated with lower mental health of pupils [7, 8]. Third, cross-sectional and longitudinal studies have found associations between key educational outcomes and mental health indicators, suggesting that mental health is an important educational resource. Analyses of large Norwegian longitudinal data show that externalising and internalising problems were associated with reductions in upper secondary school completion ranging from 33 to 54% and that this association remained significant even after controlling for family background and school characteristics [9].

In light of these arguments, a number of comprehensive intervention approaches have been developed that focus not only on individual behaviour change but also on organisational change by strengthening the wider physical and social environment and by targeting not only pupils but also teachers and parents [10–12]. However, the evidence base for these whole-school approaches is still limited and inconsistent. In their Cochrane systematic review, Langford and colleagues [13] found small intervention effects for Health Promoting School interventions on a wide range of outcomes (e.g. BMI, physical activity, fruit and vegetable consumption), but not on mental health. However, in terms of age, this refers to older children, not primary school children. In contrast, Weare and Nind’s [14] review of reviews identified small low-to-moderate effects of school-based interventions on mental health and social, emotional and academic outcomes, with greater effects for interventions with longer duration, a balance of universal and targeted approaches, use of a multimodal/whole-school approach and high implementation fidelity. In addition, findings from a meta-analysis of 45 studies showed small improvements from whole-school interventions on pupils’ social and emotional skills, externalising and internalising symptoms. The heterogeneity of these results may be explained, among other things, by the differences in methodological criteria or the range of interventions included. While the Cochrane review by Langford et al. [13] focused exclusively on cluster-randomised controlled trials examining interventions based on the Health Promoting School approach, all the other reviews also included studies using a quasi-experimental design with a comparison group or other designs. Moreover, the review by Weare and Nind [14] included a wide range of universal and targeted interventions. In terms of school type, there is also a scarcity of evidence for whole-school interventions such as MindMatters, particularly for primary schools. Existing evaluation studies for primary schools focus primarily on behavioural prevention programmes rather than on holistic interventions [15].

In summary, uncertainties remain in the evidence for whole-school approaches to mental health promotion, partly due to weak study designs. MindMatters is a comprehensive German school-based intervention that extends from primary to secondary schools and includes curriculum materials and elements aimed at developing the school ethos and environment, taking into account the wider school environment. Previous German evaluation studies have focused on secondary schools and used a questionnaire‐based pre‐post design. While teachers reported improvements in school quality, pupils were found to have lower levels of psychovegetative complaints and psychological distress after programme implementation [16, 17]. The following paper presents the study protocol for the evaluation of the primary school module of MindMatters.

### Aims and hypothesis

The study aims to replicate the intervention effects of MindMatters previously identified for primary schools [16, 17] and to examine the additional effects. Based on a confirmatory multicentre trial, a more rigorous study design will be applied, including a comparable control group. At the pupil level, the focus will be on social-emotional skills, mental health and behavioural problems. In addition to replicating the results at the pupil level, the evaluation aims to examine the changes at the organisational and classroom level, e.g. in terms of improved learning and social climate as perceived by teachers. The study will also examine whether pupils’ academic performance can be improved by strengthening their mental health.

The following hypotheses will be tested:

1. Pupils in intervention schools show higher levels of emotion-related knowledge than pupils in control schools.
2. Pupils in intervention schools have higher levels of social-emotional skills and lower levels of mental health and behavioural problems at post-test compared to pupils in control schools.
3. Teachers in intervention schools report improved diagnostic skills, improved learning and social climate and reduced classroom disruption at post-test compared to teachers in control classes.
4. Parents and teachers of pupils in intervention schools report improved academic performance and learning behaviour compared to parents and class teachers in control schools.
5. Intervention schools with higher implementation quality show greater effectiveness in terms of pupils’ social-emotional skills and mental health, and classroom outcomes such as bullying, classroom climate and disruption, compared to schools with lower implementation quality.

## Methods and analyses

### The MindMatters intervention

Originally developed in Australia in the late 1990s, MindMatters is a universal mental health promotion intervention for primary and secondary schools that takes a holistic approach to school health promotion. The German adaptation (www.mindmatters-schule.de) took place from 2002 to 2005, with 32 schools implementing the intervention modules over an 18-month period. It is based on the concept of the ‘Good and Healthy School’, which proposes a close link between health and education. This approach starts from the educational quality of the school and tries to promote this quality through health interventions. Health is thus seen as a driving force or an essential determinant of successful educational processes [18]. In line with the Health Promoting School approach, the Good and Healthy School is a holistic or whole-school approach that addresses different target groups (pupils, teachers, parents) and, in addition to individual behaviour, the wider school environment. In addition to the general promotion of mental health and the prevention of mental disorders in pupils, MindMatters aims to:

- Develop a school culture where all school members feel safe, valued and included
- Support schools in building or developing a caring school culture and networking between schools and the school environment
- Contribute to improvements in teaching and learning, thereby enhancing the quality of education in schools

For secondary schools, the intervention consists of a total of nine modules. Three of these modules are related to school development and address the development of school structures, policies and community partnerships to promote mental health. In addition, there are five subject-specific teaching modules dealing with topics such as preventing bullying, making friends, dealing with stress, mental disorders and experiences of loss and grief (Additional file 1: Overview of the German-language MindMatters intervention).

Since 2012, a separate intervention module for primary schools called ‘Learning Together with Emotions’ has been launched [19]. It supports primary schools in promoting social-emotional learning (SEL) in the classroom and is based on the five broad and interrelated domains of competence developed by the Collaborative for Academic, Social and Emotional Learning [20]. These include the following:

1. Self-awareness, i.e. the ability to understand one’s own emotions, thoughts and values
2. Self-management, i.e. the ability to manage one’s emotions, thoughts and behaviour effectively in different situations
3. Responsible decision-making, i.e. the ability to make caring and constructive choices about personal behaviour and social interactions in different situations
4. Relationship skills, i.e. the ability to establish and maintain healthy and supportive relationships and to navigate an environment with diverse individuals and groups
5. Social awareness, i.e. the ability to understand the perspectives of and empathise with others

Each competence area is covered by a teaching unit with five exercises and a final activity (e.g. a game, a song), which can be used by the teacher as a flexible resource at the curricular level (i.e. 30 activities in total). There are different variations for each exercise, which allows the intervention to be adapted to the individual needs of the pupils (different learning levels, e.g. exercises for children with and without literacy skills). Each exercise takes between 15 and 30 min. to complete, depending on the variation chosen. In addition, there are seven exercises that can be used to complement or introduce the units (e.g. setting class rules, finding each other in groups, pupils as researchers). As shown in Additional file 1, the teaching module is complemented by the SchoolMatters school development module. Designed as a basic module, SchoolMatters aims to support schools in creating structures and processes to maintain and promote mental health (e.g. conducting a needs assessment, developing a project plan, establishing a MindMatters school team, building partnerships with community stakeholders).

The intervention materials are primarily considered as a flexible resource to be used by the teacher according to the needs of the school. Although a sequence of exercises is provided within the teaching module, there is a low degree of standardisation in the implementation of the intervention. The main target group for implementation are teachers, school principals and school teams who can voluntarily attend a MindMatters training. A website (www.mindmatters-schule.de) provides training dates and access to a toolbox of further classroom exercises.

### Study design

**Fig. 1:**
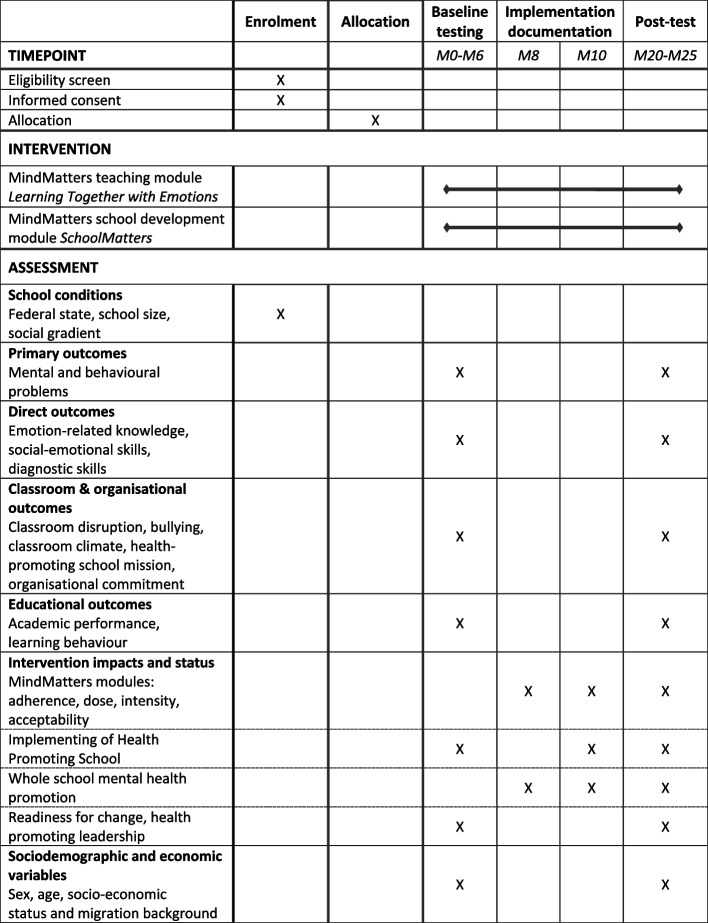
SPIRIT figure

This study is an ongoing two-arm cluster-randomised controlled superiority trial (CRCT) with baseline and post-measurement. The study protocol follows the standard protocol for clinical trials according to the SPIRIT 2013 statement (Fig. 1; Additional file 2: SPIRIT checklist) [21].

**Fig. 2:**
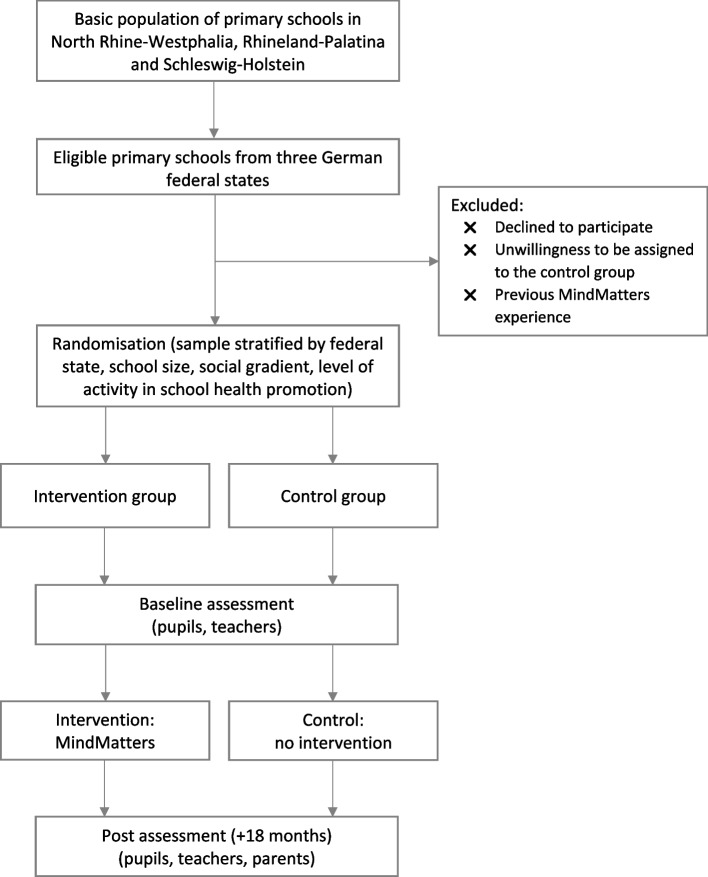
Trial flow chart

Primary schools are eligible to participate if (1) they are from one of the three federal states (North Rine-Westphalia, Rhineland-Palatinate, Schleswig–Holstein), (2) they have not implemented the MindMatters intervention in the past and (3) they agree to be assigned to either the intervention or control group. After enrolment, eligible schools will be randomly assigned 1:1 to either the intervention or control group using the Microsoft Excel random number function, with randomisation stratified by subgroups: federal state, school size, social gradient and level of activity in school health promotion. Within the subgroups, each school will be assigned a random number, with the proviso that, for example, schools with large random numbers will be assigned to the intervention group and schools with small random numbers will be assigned to the control group. Randomisation and communication with schools will be carried out by the investigators. Schools will be informed individually by e-mail of the outcome of the group allocation. Data will be collected at baseline and again 18 months after the start of the intervention, including the perspectives of pupils and teachers (both baseline and post-test) as well as parents (post-test only). While the MindMatters intervention is implemented in the intervention schools according to defined criteria, no intervention is implemented in the control schools. The trial flow of the evaluation study is shown in Fig. 2. The trial has been approved by the Ethical Review Board of Fulda University of Applied Sciences (Nr. 3.1.9.2), the Ministries of Education in Rhineland-Palatinate and Schleswig–Holstein, and registered with the German Clinical Trials Register (DRKS00023762).

### Sample size calculation

In this study, schools represent the intervention clusters in which classes and pupils are nested. The power analysis relates to the individual pupil level, taking into account the cluster effect by including the intraclass correlation (ICC). There is no ICC estimate for mental disorders in the target age group, but it is estimated to be rather low compared to substance use or risky behaviour and is therefore set at *p* = 0.01 for the power analysis [22]. While the medium effects can be expected at the level of knowledge for primary school pupils (*d* = 0.5), small effects (*d* = 0.1 to 0.3) are assumed at the level of competence due to the short study period. Given the most recent findings on the prevalence, it is expected that the prevalence of mental health problems in the intervention group will be at most four percentage points lower than in the control group at the post-test.

Assuming an alpha level of 5% and a statistical power of 0.80, the estimated sample size is 2066 (with equal allocation to both groups, i.e. 1033 pupils in the intervention group and 1033 in the control group, https://www.sealedenvelope.com/power). Taking into account an intra-class correlation of 0.01 and a number of approximately 15 pupils per class with parental consent and actually present on the day of the survey, the required total sample size increases as follows: (1 + (15 − 1) × 0.01) × 2066 = 2355. Based on previous studies in primary school education, an attrition rate of about 10% is calculated for the pre-post survey at the pupil level across the different measurement points, so that a total sample of 2615 pupils from 174 classes is required for the baseline test. It is planned to implement this with a total of 40 participating primary schools (20 in the intervention group and 20 in the control group).

### Recruitment and strategies for achieving adequate participant enrolment

As MindMatters is a complex intervention that addresses several levels, a period of 4 months is estimated for the schools to be recruited. Recruitment takes place in a total of three federal states (Rhineland-Palatinate, North Rhine-Westphalia and Schleswig–Holstein), with all primary schools being invited to participate. As a first step, all school principals will be invited to participate in the study by email. After a reminder by post, all schools will be contacted by telephone by the study team. Schools with a positive response will be contacted personally and will be informed about the process of the study. After agreeing to participate, the principal receives a short online questionnaire to assess the school’s general conditions (e.g. current implementation of school health promotion).

At the pupil level, data response is ensured by having trained academic staff collecting the data in the classroom. The success rate depends largely on the number of pupils with parental consent. To increase the response rate of parental consent forms, parents receive a detailed information letter. To increase the response rate among parents and teachers, the survey is offered both online and in paper–pencil form. The return of data is continuously monitored and reported back to schools at regular intervals. The feedback explicitly states that the significance of participating in the study is highly dependent on the level of data response. To encourage retention and completion of follow-up, schools are promised individual feedback on results at the end of the study. In addition, intervention and control schools will receive regular updates on the status of the study and motivational messages (e.g. greeting cards).

### Measures

The intervention model includes a set of outcomes that unfold gradually in the short-, medium- and long-term levels and can be observed at the school and individual levels (mainly pupils and teachers, Additional file 3: MindMatters intervention model for primary schools) [23]. The primary outcome of this study includes several indicators of pupils’ mental health (e.g. emotional symptoms, conduct problems, prosocial behaviour). According to Nutbeam’s outcome model, these effects are preceded by direct effects at the individual level, such as knowledge or skills, but also at the classroom and organisational levels, such as classroom climate, classroom disruption, integration of health promotion into the school mission or organisational commitment. In line with the basic assumption of the Good Healthy School approach, it is also assumed that improvements in direct outcomes and mental health will have a positive long-term effect on pupils’ educational outcomes (e.g. academic performance, learning behaviour). Finally, the intervention model includes a number of factors that have been shown in previous studies to be relevant to intervention effectiveness. In addition to ‘traditional’ measures of implementation fidelity, such as adherence, dosage or acceptability [24], we included further measures such as health-promoting leadership or readiness for change [25]. Table 1 provides an overview of the measurements.

**Table 1:** Overview of study measurements

Construct	Measure	Metric	Method of aggregation	Source
Mental and behavioural problems	SDQ [26]	Ordinal	Sum score	PU, T, PA
Emotion related knowledge	Self-developed	Nominal	Sum score	PU
Social-emotional skills	FEESS 1–2 [27]	Nominal	Sum score	PU
Diagnostic skills	COAKTIV [28]	4-point scale	Mean	T
Classroom disruption	FASS [29]	5-point scale	Sum score	T
Mobbing	PISA [30]	4-point scale	Sum score	T
Classroom climate	QuaSSU [31]	4-point scale	Sum score	T
Health Promoting School mission	Extended SEP scale on health promotion [32, 33]	5-point scale	Mean	T
Organisational commitment	COMMIT [34]	4-point scale	Mean	T
Academic performance	Adapted by [35]	5-point scale	Mean	T, PA
Learning behaviour	Adapted by [35]	5-point scale	Mean	T, PA
Implementation of MindMatters teaching module	Adapted by [36]	Mixed	Weighted sum-score	T
Implementation of MindMatters school development module	Self-developed	Mixed	Weighted sum score	T
Implementation of Health Promoting School	USG [37]	4-point scale	Mean	T (principals)
Whole-school mental health promotion	SSPESH [38]	4-point scale	Mean	T (principals)
Readiness for change	KESS 7, [39]	4-point scale	Mean	T
Health-promoting leadership	HoL [40]	5-point scale	Mean	T
Sex	Self-developed	Nominal		PU, T
Age	Self-developed	In years	Mean	T
Socioeconomic status (classroom level)	NEPS [41]	Percentage	Mean	T
Migration background (classroom level)	NEPS [41]	Percentage	Mean	T

*PU* pupils, *PA* parents, *T* teachers

#### Mental health outcomes

The change in mental and behavioural problems from baseline to post-assessment is measured using the German version of the Strengths and Difficulties Questionnaire (SDQ). It contains five subscales (hyperactivity, emotional symptoms, conduct problems, peer problems and prosocial scale) with five items for each dimension. In order to increase the comprehensibility and applicability for primary school children, the SDQ items have been revised, and each item has been visualised with a picture. The pupil survey is administered using an innovative tablet computer-based procedure and was pre-tested in June 2021 with 908 pupils in 10 primary schools and 43 classes in North Rhine-Westphalia.

#### Direct outcomes

Direct outcomes at the pupil level are emotion-related knowledge and social-emotional skills. Social-emotional skills were operationalised with a random selection of 14 items from the German FEESS questionnaire. The instrument consists of seven subscales, each assessed by two items (social integration, classroom climate, self-concept, attitude to school, willingness to make an effort, enjoyment of learning, feeling of acceptance). In addition, teachers’ diagnostic ability to identify conflicts and problems among pupils is measured by four items.

#### Classroom and organisational outcomes

At the classroom level, classroom disruption, e.g. due to disturbing noises, insufficient preparation by pupils or constant need for reminders, is operationalised with seven items. In addition, bullying and classroom climate (e.g. conflicts among pupils, good relationships in class) are measured with six and four items, respectively. At the organisational level, the extent to which health promotion is embedded in the school mission is assessed with eight items, while organisational commitment is operationalised with a five-item scale.

#### Educational outcomes

In terms of pupils’ educational outcomes, we assess teachers’ and parents’ reports of pupils’ academic achievement in mathematics, reading and spelling, as well as their learning behaviour, which could be rated on a 5-point scale (very good to poor).

#### Implementation

In order to assess the quality of implementation of the intervention, each MindMatters lesson or exercise delivered is documented. The documentation includes information on dose (i.e. number of exercises delivered), variation used, fidelity, deviations and preparedness. As a growing body of research suggests that school mental health interventions that expose the whole class to the same content may also have adverse effects [42, 43], teachers are asked to provide open feedback on each lesson. In this way, unintended events (e.g. harmful effects) or deviations are assessed in a non-systematic approach.

In addition to the activities implemented at the curriculum level, teachers from intervention schools are asked to document their school development activities to maintain and promote mental health as defined in the SchoolMatters intervention module (degree of engagement with the SchoolMatters materials, establishment of a school development team, conducting a needs assessment, creating a project plan and a plan for crisis situations, publicity, raising awareness of the intervention within and outside the school) and to assess satisfaction with support and other feedback.

#### Covariates, mediators and moderators

For both intervention and control schools, it will be recorded whether other mental health interventions were implemented during the study period. Furthermore, both groups will provide information on health promotion activities, school development, readiness to change and health-promoting leadership. Sociodemographic characteristics (sex, age), socioeconomic status and migration background of participants will also be recorded.

#### Data collection, management and statistical analysis

IFT Nord is responsible for the coordination and monitoring of the study. Two study centres (IFT-Nord, Fulda University of Applied Sciences) are responsible for the collection, processing and use of the data. In both study centres, all data will be collected using the same procedures, standards and elaborated instruments of proven psychometric quality. Three different sources of data are used: (1) online survey data from school principals, teachers and parents; (2) pupil survey data collected at classroom level via tablet computers; and (3) paper–pencil questionnaire data from principals, teachers and parents. All data will be stored on a secure IT infrastructure. Data from paper-based questionnaires are stored in a lockable cabinet and are only removed temporarily for data entry. A data safety concept is in place that has been reviewed and monitored by the Ministries of Education of the participating study regions. Data entry is carried out by trained scientific staff and is randomly checked by a second independent researcher. Once collected and entered, all data will be checked for plausibility, consistency and completeness. Data transfer between the two study centres is ensured via a secure connection (e.g. virtual private network (VPN)). The status of the trial will be documented in regular progress reports and by the Federal Ministry of Education and Research. To ensure quality, regular site visits will be organised at the study centres.

Statistical analyses will be performed using the latest version of STATA (Stata Corp., College Station, TX, USA). The main analysis will consist of a multilevel regression in which the primary outcomes are predicted at the pupil level using group status (intervention versus control group), controlled for baseline condition. Group status will be included in the model as a fixed effect and a random intercept will be provided for the cluster variables (study region, school, class). Furthermore, all variables with differences between the intervention and control group at baseline will be included in the model. Further mediation analyses will investigate which mediating mechanisms can be identified. It is assumed that the change in mental health outcomes is caused by improvements in proximal (i.e. direct and class/organisational) outcomes. This requires that the group status (i.e. the intervention) is correlated with the proximal outcomes and that the proximal outcomes are significantly related to the distal outcomes. Mediation effects will be examined using the Baron and Kenny partial correlation analysis approach and structural equation models [44, 45]. Moderator analyses will also examine which school-level factors (e.g. size, social gradient) are associated with or influence the impact of MindMatters. Moderator effects are tested using interaction terms in the regression model. The analysis will first be conducted according to the intention-to-treat principle and additionally compared with a per-protocol analysis. Analysis sample 1 includes all those for whom data are available at both measurement points (complete case analysis, as randomised). Analysis sample 2 includes all those reached at baseline (ITT as randomised). Analysis sample 3 includes all those with data available at both measurement points, and schools without intervention are included in the control group. Multiple imputation is used for missing values. To ensure the methodological quality of all analyses, statistical advice is provided by an independent institute.

## Discussion

This study protocol documents the two-arm cluster randomised controlled trial with baseline and post-assessment for MindMatters primary schools, a holistic school-based intervention to promote mental health in school children. While the overall intervention targets children and adolescents in primary and secondary schools, this study focuses on the primary school module, for which there is no previous evidence. Reductions in mental and behavioural problems in children will be the primary outcomes, preceded by improvements in knowledge and social-emotional skills, as well as organisational improvements such as classroom climate, classroom disruption and organisational commitment. In addition, we expect these intervention effects to have a positive long-term impact on pupils’ educational outcomes.

While the effectiveness of highly standardised behavioural modification interventions is well documented, there is still an evidence gap for complex intervention programmes that take into account environmental and organisational determinants. This problem has also been labelled as ‘inverse evidence law’ [23], and the results of this study aim to contribute to strengthen the evidence base for holistic interventions on school (mental) health promotion. Its strengths can be seen in the study design (a two-arm cluster randomised controlled trial), which has a comparatively high internal validity. By interviewing younger children, the study focuses on a target group that has received little attention so far; to date, parents or teachers have mostly been interviewed, but not the children themselves [46]. Younger children’s own perspectives can provide valuable information for assessing mental health as part of a multi-informant approach [47]. In addition, innovative methods are used: pupils are surveyed using tablet computers that guide them through the questionnaire in the classroom in a structured and visually appealing way. The inclusion of pictorial representations of the questions may improve the reliability of self-reports in younger children [48]. It should also be emphasised that the study links several indicators of implementation fidelity with intervention effectiveness and will also help to shed more light on the relationship between health and educational attainment. Evidence from the Wales-wide School Health Research Network suggests that school health policies and curriculum practices are associated with educational attainment at age 14, particularly in disadvantaged schools [49]. However, as emphasised by Langford et al. [13] in their Cochrane review, the available evidence is scarce and does not allow conclusions to be drawn about the effectiveness of interventions on school health promotion. Evidence of such effects would also confirm the conceptual approach of the Good Healthy School [18]. As health is not a mandatory part of the school curriculum or cross-curricular activities in many countries, evidence would provide important arguments for establishing (mental) health in everyday school life. Finally, it should be noted that the results of the trial also aim at helping funding institutions and decision-makers in allocating resources.

The main limitation of this study is the assessment via self-reports. There is an increased risk of systematic bias, such as recall bias. Especially in younger children, challenges such as limited language and cognitive abilities or comprehension difficulties must be taken into account [50], although self-reports have previously been shown to be reliable and valid measures of mental well-being in adolescents. In addition, the power calculation was made a priori without information on the ICC estimate for mental disorders in children. Post hoc, the ICC for the SDQ could be determined from the pilot data and it was found that the ICCs ranged from *p* = 0.03 (hyperactivity) to *p* = 0.07 (behavioural problems with peers), indicating a severe underestimation of the ICCs for the SDQ. Furthermore, the SDQ might not be an adequate proxy for mental disorders, as it includes, for example, items for classroom disruption and prosocial behaviour. Although the trial might therefore not be suitably powered to detect small intervention effects, type I error inflation might be cushioned by the large number of clusters at the classroom level.

Also, as participation is voluntary, non-compliance could become an issue. Teachers are expected to teach the prepared modules and excercises independently of their teaching responsibilities and may find this an additional burden. These classes may discontinue participation in MindMatters early. Another limitation to the implementation of the intervention is the significant additional burden placed on schools by the Corona pandemic. As health promotion is not legally anchored in schools, this could lead to limited resources being invested in school health interventions [51]. As a result, a certain selection bias in school participation cannot be ruled out. Finally, this study is conducted in three German federal states, and conclusions cannot be directly transferred to other federal states due to the differences in the education systems.

### Trial status

Protocol version 2, 6 October 2023. Recruitment of schools started in May 2021 and was completed in January 2022, with data collection continuing until September 2023. Due to the Corona pandemic, the data collection process had to be adapted several times.

### Supplementary Information

**Additional file 1. **Overview of the German-language MindMatters intervention.

**Additional file 2. **SPIRIT checklist.

**Additional file 3. **MindMatters intervention model for primary schools.

## Data Availability

Publications will be made open access. The datasets generated during the current study will not be made open access but may be made available on request to the principal investigators (KD, MM) and according to the ethical approval. At present, there are no plans to use the data in ancillary studies, and no consent has been obtained from participants.

## Abbreviations

COAKTIV: Cognitive Activation in the Classroom: The Orchestration of Learning Opportunities for the Enhancement of Insightful Learning in Mathematics
COMMIT: Commitment-Skalen [Commitment Scales]
COPSY: Corona und Psyche [Corona and Mind]
CRCT: Cluster-randomised controlled trial
*d*: Cohen’s *d* effect size
DALY: Disability-adjusted life years
FASS: Fragebogen zur Arbeitssituation an Schulen [questionnaire on the working situation in schools]
FEESS: Fragebogen zur Erfassung emotionaler und sozialer Schulerfahrungen [questionnaire for the assessment of emotional and social school experiences]
GBD: Global Burden of Disease
GHQ-12: General Health Questionnaire
HoL: Health-oriented leadership
ICC: Intraclass correlation
IFT Nord: Institute for Therapy and Health Research North
KESS: Kompetenzen und Einstellungen von Schülerinnen und Schülern [competencies and attitudes of pupils]
NCD: Non-communicable diseases
NEPS: National Educational Panel Study
PISA: Programme for International Student Assessment
QuaSSU: Qualitätssicherung in Schule und Unterricht [quality assurance in school and teaching]
SDQ: Strengths and Difficulties Questionnaire
SEL: Social-emotional learning
SEP: Selbstevaluationsportal [self-evaluation portal]
SPIRIT: Standard Protocol Items: Recommendations for Interventional Trials
SSPESH: Survey of School Promotion of Emotional and Social Health
STATA: Statistical Software for Data Science
USG: Umsetzungsstand schulischer Gesundheitsförderung [implementation status of school health promotion]
VPN: Virtual private network

## References

[1] Patton GC, Sawyer SM, Santelli JS, Ross DA, Afifi R, Allen NB, Arora M, Azzopardi P, Baldwin W, Bonell C, Kakuma R, Kennedy E, Mahon J, McGovern T, Mokdad AH, Patel V, Petroni S, Reavley N, Taiwo K, Waldfogel J, Wickremarathne D, Barroso C, Bhutta Z, Fatusi AO, Mattoo A, Diers J, Fang J, Ferguson J, Ssewamala F, Viner RM (2016). Our future: a Lancet commission on adolescent health and wellbeing. The Lancet.

[2] Baranne ML, Falissard B (2018). Global burden of mental disorders among children aged 5–14 years. Child Adolesc Psychiatry Ment Health.

[3] Polanczyk GV, Salum GA, Sugaya LS, Caye A (2015). Annual research review: a meta‐analysis of the worldwide prevalence of mental disorders in children and adolescents. J Clin Child Psychol.

[4] Silva SA, Silva SU, Ronca DB, Goncalves VSS, Dutra ES, Carvalho KMB (2020). Common mental disorders prevalence in adolescents: a systematic review and meta-analyses. PLoS ONE.

[5] Schlack R, Neuperdt L, Junker S, Eicher S, Hölling H, Thom J, Ravens-Sieberer U, Beyer AK (2023). Changes in mental health in the German child and adolescent population during the COVID-19 pandemic. Results of a rapid review. J Health Monit.

[6] Ravens-Sieberer U, Erhart M, Devine J, Gilbert M, Reiss F, Barkmann C, Siegel NA, Siimon AM, Hurrelmann K, Schlack R, Hölling H, Wieler LH, Kaman A (2022). Child and adolescent mental health during the COVID-19 pandemic: results of the three-wave longitudinal COPSY study. J Adolesc Health.

[7] László KD, Andersson F, Galanti MR (2019). School climate and mental health among Swedish adolescents: a multilevel longitudinal study. BMC Public Health.

[8] Milkie MA, Warner CH (2011). Classroom learning environments and the mental health of first grade children. J Health Soc Behav.

[9] von Simson K, Brekke I, Hardoy I (2022). The impact of mental health problems in adolescence on educational attainment. Scand J Educ Res.

[10] Dadaczynski K, Jensen BB, Grieg-Viig N, Sormunen M, von Seelen J, Kuchma V, Vilaca T (2020). Health, well-being and education: building a sustainable future. The Moscow statement on Health Promoting Schools. Health Educ.

[11] Margaretha M, Azzopardi PS, Fisher J, Sawyer SM (2013). School-based mental health promotion: a global policy review. Front Psychiatry.

[12] World Health Organization (WHO) (2021). Mental health in schools: a manual. Cairo: World Health Organization. Regional Office for the Eastern Mediterranean; 2021.

[13] Langford R, Bonell C, Jones H, Pouliou T, Murphy S, Waters E, Komro K, Gibbs L, Magnus D, Campbell R (2015). The World Health Organization’s Health Promoting Schools framework: a Cochrane systematic review and meta-analysis. BMC Public Health.

[14] Weare K, Nind M (2011). Mental health promotion and problem prevention in schools: what does the evidence say?. Health Promot Int.

[15] Fenwick-Smith A, Dahlberg EE, Thompson SC (2018). Systematic review of resilience-enhancing, universal, primary school-based mental health promotion programs. BMC Psychol.

[16] Franze M, Paulus P (2009). MindMatters - a programme for the promotion of mental health in primary and secondary schools. Results of an evaluation of the German language adaptation. Health Educ.

[17] Franze M, Meierjürgen R, Abeling I, Rottländer M, Gerdon R, Paulus P (2007). indMatters. Ein Programm zur Förderung der psychischen Gesundheit in Schulen der Sekundarstufe 1 - deutschsprachige Adaptation und Ergebnisse des Modellversuchs. Präv Gesundheitsf.

[18] Paulus P, Jensen BB, Clift S (2005). From the health promoting school to the good and healthy school: new developments in Germany. The health promoting school: international advances in theory, evaluation and practice.

[19] Nieskens B, Heinold F, Paulus P. MindMatters. Gemeinsam(es) Lernen mit Gefühl. Eine Ressource zur Förderung sozial-emotionaler Kompetenzen in der Primarstufe. Lueneburg: Leuphana Universität Lueneburg; 2011.

[20] Borowski R. CASEL’s Framework for Systemic Social and Emotional Learning. Measuring SEL: using data to inspire practice. Chicago, IL: Collaborative for Academic. Social, and Emotional Learning. 2019. Retrieved from: https://measuringsel.casel.org/wp-content/uploads/2019/08/AWG-Framework-Series-B.2.pdf. Accessed 02 June 2023

[21] Chan A-W, Tetzlaff JM, Gøtzsche PC, Altman DG, Mann H, Berlin J, Dickersin K, Hróbjartsson A, Schulz KF, Parulekar WR, Krleža-Jerić K, Laupacis A, Moher D (2013). SPIRIT 2013 Explanation and Elaboration: guidance for protocols of clinical trials. BMJ.

[22] Shackleton N, Jamal F, Viner RM, Dickson K, Patton G, Bonell C (2016). School-based interventions going beyond health education to promote adolescent health: systematic review of reviews. J Adolesc Health.

[23] Nutbeam D (1998). Evaluating health promotion - progress, problems and solution. Health Promot Int.

[24] Dusenbury L, Brannigan R, Falco M, Hansen WB (2003). A review of research on fidelity of implementation: implications for drug abuse prevention in school settings. Health Educ Res.

[25] Samdal O, Rowling L (2011). Theoretical and empirical base for implementation components of health-promoting schools. Health Educ.

[26] Goodman R (1997). The Strengths and Difficulties Questionnaire: a research note. J Child Psychol Psychiatry.

[27] Rauer W, Schuck KD. FEESS 1–2. Fragebogen zur Erfassung emotionaler und sozialer Schulerfahrungen von Grundschulkindern erster und zweiter Klassen. Göttingen: Beltz; 2004.

[28] Baumert J, Blum W, Brunner M, Dubberke T, Jordan A, Klusmann U, Krauss S, Kunter M, Löwen K, Neubrand M, Tsai YM (2009). Professionswissen von Lehrkräften, kognitiv aktivierender Mathematikunterricht und die Entwicklung von mathematischer Kompetenz (COACTIV) Dokumentation der Erhebungsinstrumente.

[29] Krause A. Fragebogen zur Arbeitssituation an Schulen (FASS). Dokumentation der Skalen und Aussagen. Freiburg: Albert-Ludwigs Universität Freiburg. 2004. Retrieved from: https://tinyurl.com/34n49bjs. Accessed 24 Jul 2023

[30] Konsortium PISA.ch. PISA 2018: Schülerinnen und Schüler der Schweiz im internationalen Vergleich. Bern & Genf: SBFI/EDK und Konsortium PISA.ch; 2019.

[31] Ditton H, Merz D (2000). Qualität von Schule und Unterricht.

[32] Institut für Schulqualität der Länder Berlin und Brandenburg. Selbstevaluationsportal. Schule: Modul Gesundheitsförderung. 2019. Retrieved online: https://tinyurl.com/46av7smv. Accessed 28 Feb 2020

[33] Harazd B, Gieske M, Rolff HG. Gesundheitsmanagement in der Schule: Lehrergesundheit als neue Aufgabe der Schulleitung. Köln: LinkLuchterhand; 2009.

[34] Felfe J, Franke F. Commitment–Skalen (COMMIT). Fragebogen zur Erfassung von Commitment gegenüber Organisationen, Beruf/Tätigkeit, Team, Führungskraft und Beschäftigungsform. Manual. Bern: Hans Huber; 2012.

[35] Husky MM, Salamon R, Bitfoi A, Carta MG, Chan Chee C, Goelitz D, Pez O (2020). Self-reported mental health problems and performance in mathematics and reading in children across Europe. Eur J Dev Psychol.

[36] Isensee B, Hanewinkel R. Klasse 2000: Evaluation des Unterrichtsprogramms in Hessen. Abschlussbericht. Kiel: IFT-Nord, Institut für Therapie- und Gesundheitsforschung; 2009.

[37] Dadaczynski K, Hering T (2021). Health promoting schools in Germany. Mapping the implementation of holistic strategies to tackle NCDs and promote health. Int J Environ Res Public Health.

[38] Dix K, Green MJ, Tzoumakis S, Dean K, Harris F, Carr VJ, Laurens KR (2018). The Survey of School Promotion of Emotional and Social Health (SSPESH): a brief measure of the implementation of whole-school mental health promotion. Sch Ment Health.

[39] Bos W, Bonsen M, Gröhlich C, Guill K, Scharenberg K. KESS 7 - Skalenhandbuch zur Dokumentation der Erhebungsinstrumente. Hanse - Hamburger Schriften zur Qualität im Bildungswesen. Münster: Waxmann; 2009.

[40] Pundt F, Felfe J (2017). Health-oriented Leadership HoL – Instrument zur Erfassung gesundheitsförderlicher Führung.

[41] Blossfeld HP, Roßbach HG, von Maurice J, editors. Education as a lifelong process. The German National Educational Panel Study (NEPS). Wiesbaden: VS Verlag für Sozialwissenschaften; 2011.

[42] Foulkes L, Stringaris A (2023). Do no harm: can school mental health interventions cause iatrogenic harm?. BJPsych Bulletin.

[43] Foulkes L, Stapley E (2022). Want to improve school mental health interventions? Ask young people what they actually think. J Philos Educ.

[44] Baron RM, Kenny DA (1986). The moderator-mediator variable distinction in social psychological research: conceptual, strategic, and statistical considerations. J Pers Soc Psychol.

[45] Hayes AF (2009). Beyond Baron and Kenny: statistical mediation analysis in the new millennium. Commun Monogr.

[46] De Los Reyes A, Augenstein TM, Wang M, Thomas SA, Drabick DAG, Burgers DE, Rabinowitz J (2015). The validity of the multi-informant approach to assessing child and adolescent mental health. Psychol Bull.

[47] Deighton J, Croudace T, Fonagy P, Brown J, Patalay P, Wolpert M (2014). Measuring mental health and wellbeing outcomes for children and adolescents to inform practice and policy: a review of child self-report measures. Child Adolesc Psychiatry Ment Health.

[48] Kuijpers RC, Otten R, Vermulst AA, Pez O, Bitfoi A, Carta M, Goelitz D, Keyes K, Koç C, Lesinskiene S, Mihova Z, Engels RC, Kovess V (2016). Reliability, factor structure, and measurement invariance of the Dominic Interactive across European countries: cross-country utility of a child mental health self-report. Psychol Assess.

[49] Long SJ, Littlecott H, Hawkins J, Eccles G, Fletcher A, Hewitt G, Murphy S, Moore GF (2020). Testing the ‘zero-sum game’ hypothesis: an examination of school health policies and practices and inequalities in educational outcomes. J Sch Health.

[50] Marsh HW, Debus R, Bornholt LJ (2008). Validating young children’s self-concept responses: methodological ways and means to understand their responses. In: Teti DM, editor. Handbook of Research Methods in Developmental Science. Oxford: John Wiley & Sons; 2008, p. 138–160

[51] Dadaczynski K, Okan  O, Messer M (2022). Schulische Gesundheitsförderung in pandemischen Zeiten. Ergebnisse der COVID-HL Schulleitungsstudie. Bundesgesundheitsblatt Gesundheitsforschung Gesundheitsschutz.

