# Supplementary material for: Effectiveness of the holistic primary school-based intervention MindMatters: study protocol for a cluster-randomised controlled trial
Source: Trials. 2023 Nov 8;24:711. doi: 10.1186/s13063-023-07731-0 (PMC10631112; doi:10.1186/s13063-023-07731-0)
Supplement: Supplementary file 1 — Additional file 1. Overview of the German-language MindMatters intervention. [file 13063_2023_7731_MOESM1_ESM.docx]

Supplement 1: Overview of the German-language MindMatters intervention (blue: teaching modules, orange: school development modules), www.mindmatters-schule.de

**Learning together with emotions**

A resource for promoting
 social-emotional competencies in primary school

**Soul support**

Dealing with
loss & grief

**CommunityMatters**
Opening schools so they can link with their environment

**Bullying? Not in our school**

Prevention & action strategies

**LifeMatters**
Guidelines for schools, for preventing
self-inflicted injury & suicide

**SchoolMatters**
Creating good schools with healthy minds

**Finding & keeping friends**

Promoting resilience in schools

**How are you?**

Understanding mental health problems in schools

**Coping with stress**

Promoting resilience in schools

**Fit for training and career**

Managing the transition with mental health

**Good and Healthy School**

**Primary School**

**Secondary School**
