# Supplementary material for: Effectiveness of the holistic primary school-based intervention MindMatters: study protocol for a cluster-randomised controlled trial
Source: Trials. 2023 Nov 8;24:711. doi: 10.1186/s13063-023-07731-0 (PMC10631112; doi:10.1186/s13063-023-07731-0)
Supplement: Supplementary file 3 — Additional file 3. MindMatters intervention model for primary schools. [file 13063_2023_7731_MOESM3_ESM.docx]

Supplement 3: MindMatters intervention model for primary schools

**MindMatters**

**School development
module**
SchoolMatters

**Teaching module**
Learning together with emotions

**Direct Outcomes**

- Knowledge
- Social-emotional competencies
- Diagnostical competence

**Class & organisational Outcomes**

- Classroom disruption
- Mobbing
- Class climate
- Health promoting school mission
- Organisational commitment

**Mental Health Outcomes**

- Emotional symptoms
- Conduct problems
- Peer-relationship problems
- Hyperactivity/inattention
- Prosocial behaviour

**Educational Outcomes**

- Academic performance
- Learning behaviour

**Implementation influences**

- Health promoting leadership
- Readiness for change
- Implementation fidelity
- Dosage
- Acceptance

**Input**

**Outcome**

**Implem-entation**
